# Prevalence of osteoporosis in patients with type 2 diabetes mellitus in the Chinese mainland: A protocol of systematic review and meta-analysis

**DOI:** 10.1097/MD.0000000000019762

**Published:** 2020-04-17

**Authors:** Yuhao Si, Cenyi Wang, Yang Guo, Heng Yin, Yong MA

**Affiliations:** a The First School of Clinical Medicine, Laboratory for New Techniques of Restoration & Reconstruction of Orthopedics and Traumatology, Nanjing University of Chinese Medicine, Nanjing; b Department of Rehabilitation, The First Affiliated Hospital of Guangxi University of Chinese Medicine, Nanning, China; c Rangos School of Health Sciences, Duquesne University, Pittsburgh, USA; d Affiliated Hospital of Nanjing University of Chinese Medicine, Department of Traumatology & Orthopedics, Nanjing; e Department of Traumatology & Orthopedics, Wuxi Affiliated Hospital of Nanjing University of Chinese Medicine, Wuxi; f College of Basic Medicine, Nanjing University of Chinese Medicine, Nanjing, China.

**Keywords:** mainland China, osteoporosis, prevalence, risk factors, systematic review, type 2 diabetes mellitus

## Abstract

**Background:**

Osteoporosis, as a metabolic disease, has become one of the most rapidly growing complications of diabetes mellitus. Previously, we conducted a systematic review to report the estimated prevalence of osteoporosis among patients with type 2 diabetes mellitus (T2DM) from 2001 to 2016. However, some newer studies have been reported, and more potential risk factors for osteoporosis remain controversial and vague. Therefore, the presented review is tailored to synthesize available evidence regarding the pooled prevalence and related risk factors of osteoporosis among T2DM patients in mainland China.

**Methods:**

Both cross-sectional study and cohort study reporting on the prevalence of osteoporosis among patients with T2DM in mainland China, published in Chinese or English, will be considered for inclusion. Records from 4 English databases and 2 Chinese databases will be retrieved from their inceptions to March 2020. The process of study selection, data extraction, and methodologic quality assessment will be independently executed by 2 reviewers. The pooled prevalence of osteoporosis and the prevalence in the exposed and unexposed groups for each potential risk factor will be calculated by the software Stata version 12.0.

**Results:**

The current systematic review and meta-analysis will provide more precise prevalence rates and associated risk factors of osteoporosis among people with T2DM in mainland China.

**Conclusion:**

The study will offer high-quality and explicit evidence for assisting diabetic patients to prevent osteoporosis in mainland China.

**OSF Registration:**

DOI 10.17605/OSF.IO/5ZKJ6.

## Abstract 2

Supplemental Digital Content is available in the text

## 1 Introduction

In 2015, there were approximately 415 million people with diabetes globally, and this number is projected to grow to 642 million by 2040.^[1]^ China has the largest population suffering from type 2 diabetes mellitus (T2DM) worldwide, with approximately 136.1 million in 2013, accounting for more than one-third of the global burden of diabetes mellitus.^[2,3]^ In mainland China, T2DM has become a national epidemic disease from a rare disease in the past 40 years, growing from 0.67% to 10.4%.^[4]^ Accordingly, with the elderly population increasing, the number of T2DM patients in mainland China is anticipated to rise to 150.7 million by 2040.

As is commonly known, diabetes mellitus can impair the function and condition of multiple viscera and systems in the human body,^[5–10]^ contributing to a variety of diseases, such as coronary artery disease, peripheral vascular disease, and neuropathy.^[11]^ Therefore, diabetes mellitus is currently deemed as the disease that has the most number of complications (more than 100 kinds).^[12]^ Furthermore, a concept has been realized that a potential relationship seems to exist between diabetes (mainly T2DM) and osteoporosis.^[13,14]^ However, this concept remains controversial over the risk of osteoporosis and its clinical significance in diabetic patients. People might consider that T2DM is correlated with higher bone mineral density (BMD), which is supposed to lower the risk of fracture occurrence among the ordinary population.^[15]^ Nevertheless, some recent studies suggested that T2DM patients have lower BMD and increased fracture risk, especially hip and vertebral fractures.^[16–21]^ Thus, for early clinical prevention, it is necessary to investigate the prevalence and related risk factors of osteoporosis among T2DM patients. Nonetheless, it is nearly impossible to conduct such a representative survey across the whole nation since China has a substantial population and vast territory. No systematic review with respect to the prevalence rates of osteoporosis among T2DM patients in mainland China has been implemented except for our previous meta-analysis, which covered and analyzed the studies from 2001 to 2016.^[22]^ Our findings illustrated that the pooled prevalence rate of osteoporosis among T2DM patients in mainland China was 37.8%. Notably, females or older adults with T2DM required early clinical prevention as they were more vulnerable to osteoporosis (female: 44.8% vs male: 37.0%; over 60: 40.1% vs below 60: 26.5%). Additionally, the pooled prevalence of osteoporosis was higher in less developed regions than in developed regions.

The associated risk factors for osteoporosis among Chinese T2DM patients have not been apparent, although our early study investigated several factors (gender, age, economic status, and region). Other potential risk factors, such as ethnic groups, body mass index (BMI), diet, habits, blood glucose level, glycosylated hemoglobin (GHb), triglyceride, course of the disease, and medication, remain controversial and inconclusive and therefore need to be assessed in a systematic and evidence-based approach. In consideration of the practicability for modeling a predictive model, precise estimates of relevant risk factors for osteoporosis among Chinese diabetics warrant further investigation. Besides, further study would enable the development of a tool to estimate and predict the risks of osteoporosis occurrence for diabetic patients, which is useful to individually focused management.

Due to China's recent ageing population, more attention has been paid to the epidemic of senile illness, including T2DM and osteoporosis. Some latest and high-quality studies of epidemiological investigation have been executed in mainland China, which offer us new data for directing an updated systematic review and meta-analysis for the prevalence and correlated factors of osteoporosis among Chinese diabetics.^[23–26]^ Accordingly, the present study is designed to critically synthesize the updated published data to estimate a more accurate prevalence rate of osteoporosis among Chinese diabetics and screen the associated risk factors, to offer basic knowledge for tailoring cost-effective prevention strategies of factors management. We will concentrate on the variation in the prevalence of osteoporosis for various risk factors, such as ethnic groups, BMI (obesity), medication, and disease-related molecular biomarkers, which differs from our previous review.

## 2 Methods

The current systematic review and meta-analysis have been registered in the Open Science Framework (OSF) with an identification number of DOI 10.17605/OSF.IO/5ZKJ6. The possible modification after publication will also be disclosed in the OSF. We will direct the study according to the meta-analysis of observational studies in epidemiology (MOOSE) guidelines and the preferred reporting items for systematic reviews and meta-analyses (PRISMA) statement.^[27,28]^

### 2.1 Inclusion criteria

Studies that meet the following criteria will be included in our review:

1. Participants from mainland China diagnosed with T2DM.
2. Study design being a cross-sectional study or cohort study.
3. Equipment utilized to test BMD should be dual-energy X-ray absorptiometry.
4. Tested area is lumbar or hip.
5. Outcomes are pooled prevalence estimates and the prevalence rate for each risk factor.
6. Published in English or Chinese.

### 2.2 Exclusion criteria

Studies that meet the following criteria need to be excluded from our review:

1. Participants diagnosed with other types of diabetes mellitus.
2. Review, case report, letter, or abstract.
3. A duplicate report.
4. The information regarding prevalence estimates and risk factors cannot be directly extracted or obtained from the corresponding author.

### 2.3 Search strategy

Four English databases (PubMed, Scopus, Web of Science, Cochrane Library) and 2 Chinese databases (China National Knowledge Infrastructure, Wanfang Database) will be retrieved from their inceptions to March 2020. The electronic search strategy is accessible in Supplemental Digital Content (Appendix 1). Moreover, we will also conduct a manual search in the library of Nanjing University of Chinese Medicine and Duquesne University in case of any missing literature.

### 2.4 Selection of studies

**Figure 1:**
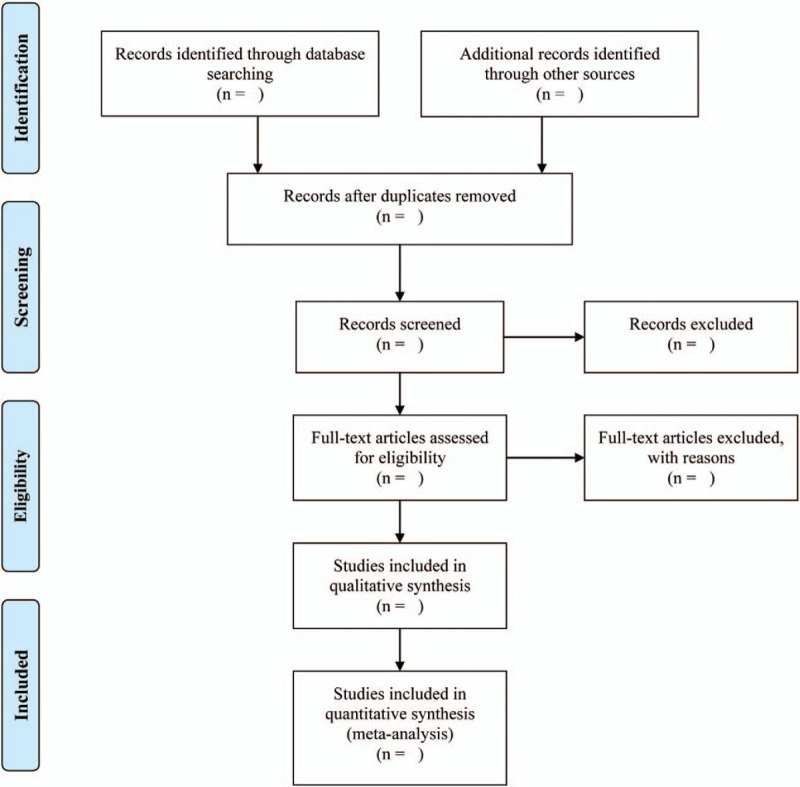
Study selection flow diagram.

Two members (Si and Wang) of our workgroup will independently screen the titles and abstracts of potential articles, then read the whole contents to further identify the eligible studies by employing the drafted inclusion and exclusion criteria. A third reviewer (Ma) will be an arbitrator to make a judgment when disputes arise. Additionally, the 2 reviewers (Si and Wang) will record the results of selection (authors, titles, and publication year) in a Microsoft Excel spreadsheet (Microsoft Corporation, Redmond, WA), and another reviewer (Yin) will supervise the whole process. The Preferred Reporting Items for Systematic Reviews and Meta-Analyses Flowchart (Fig. 1) will be filled in by the end of the study selection to provide specific information.

### 2.5 Data extraction and management

The 4 aspects of data will be extracted from the eligible studies: characteristics of studies (e.g., the first author, publication year, study design, the equipment for BMD assessment, the tested body parts, the place where the study was conducted), participants (e.g., sample size, response rate, age, gender, BMI, type and duration of diabetes, place of residence, living habits), biomarkers (e.g., level of blood glucose, GHb, triglyceride), results (overall number of osteoporosis cases, stratified number of osteoporosis cases by each factor). Two reviewers (Si and Wang) will create a spreadsheet to save all the extracted data as mentioned, and another reviewer (Yin) will supervise the whole process. If some necessary data cannot be found or calculated from the potential studies, we will email or call the corresponding author to ask for assistance. Furthermore, the study will be excluded if the missing data cannot be acquired after contacting the corresponding author.

### 2.6 Assessment of risk of bias in individual study

Two reviewers (Si and Wang) will independently assess the risk of potential bias in each study applying the Newcastle-Ottawa Scale (NOS), which is devised for nonrandomized studies.^[29]^ The NOS consists of 3 aspects, which are the selection of study groups, comparability, and assessment of outcome. Based on the 3 aspects, a total score ranging from 0 to 9 points will be measured, and a study with a total score of greater than 7 points is defined as a high-quality study. Any disagreement will be determined by a third reviewer (Yin).

### 2.7 Data analysis and synthesis

We will conduct this study on the basis of at least 2 studies employing the software Stata version 12.0. We will display all the information extracted from the eligible studies via plotting summary tables, flow diagrams, or graphs, depending on the necessities. It should be noted that we prefer to choose the random-effect model to calculate the pooled prevalence estimates since the prevalence estimates tend to vary among different populations. The heterogeneity among studies will be quantified via applying the *I*^2^ index.^[30]^ Moreover, a narrative synthesis will be performed when the meta-analysis is not suitable to be administered.

### 2.8 Publication bias

The Begg's test, as well as the Egger's test, will both be utilized to judge the potential publication bias. Additionally, the trim and fill method will be employed if the results of the Begg's test and the Egger's test demonstrate that substantial publication bias exists.

### 2.9 Subgroup analysis

It is essential to conduct the subgroup analysis in this study in order to screen the potential osteoporosis-related risk factors among Chinese diabetics. The subgroup analyses will be executed in accordance with the various types of participants (e.g., ethnic groups, BMI, age), regions (e.g., economics status, living habits, medical resources, climate) and some other information regarding the study design (e.g., year and sample size).

### 2.10 Patient and public involvement

No patients will be involved in this systematic review and meta-analysis.

### 2.11 Ethics and dissemination

There is no need for ethics approval since the current review is a synthesis of secondary public data that will not leak any personal information or violate privacy rights. The current review is supposed to offer more accurate estimated prevalence rates of osteoporosis in the diabetic population in mainland China and detect associated risk factors. The meaning of our findings is to inform the national health departments and policymakers to tailor comprehensive prevention strategies to control osteoporosis in high-risk patients with T2DM. Hopefully, our review will be published in the peer-reviewed journal and presented at academic conferences. Additionally, we will continue to update our review if new evidence is published. The study protocol will be revised in the OSF website if our research plan is modified.

## 3 Discussion

With the prevalence of T2DM in mainland China rising rapidly in a short period, increasingly more diabetics are suffering from osteoporosis. In the refreshed systematic review and meta-analysis, we will deepen our research by incorporating the latest studies and examine more individual and non-individual risk factors. Additionally, this study will continue to present the merged data concerning the prevalence rates of osteoporosis among T2DM patients in mainland China, which is conducive to formulating and optimizing national strategies to prevent osteoporosis in people living with diabetes. More importantly, the updated review intends to screen disease-related risk factors in a more accurate and broader approach, as previously mentioned. The Department of Health may adopt these evidence-based factors to construct a tool or a data model to infer the probability of osteoporosis occurrence in a particular T2DM patient.

## Acknowledgments

Hereby, we would like to express our gratitude to Professor Xi Huang, the “Outstanding Youth” at Nanjing University of Chinese Medicine, for his financial support from the project of integration of Chinese and western medicine.

## Author contributions

All the authors have critically read and approved the final version of this manuscript.

**Conceptualization**: Heng Yin, Yong Ma.

**Formal analysis:** Yuhao Si, Cenyi Wang.

**Funding acquisition**: Heng Yin, Yong Ma.

**Methodology:** Yuhao Si, Cenyi Wang, Yang Guo, Heng Yin, Yong Ma.

**Software:** Yuhao Si, Yuhao.

**Supervision**: Heng Yin, Yong Ma.

**Validation:** Heng Yin, Yong Ma.

**Writing – original draft**: Yuhao Si, Cenyi Wang.

**Writing – review & editing**: Heng Yin, Yong Ma.

## Supplementary Material

Supplemental Digital Content
