# Supplementary material for: Prevalence of osteoporosis in patients with type 2 diabetes mellitus in the Chinese mainland: A protocol of systematic review and meta-analysis
Source: Medicine (Baltimore). 2020 Apr 17;99(16):e19762. doi: 10.1097/MD.0000000000019762 (PMC7220203; doi:10.1097/MD.0000000000019762)
Supplement: Supplemental Digital Content [file medi-99-e19762-s001.docx]

**Appendix 1. Search strategy for PubMed**

| No. | Search terms |
| --- | --- |
| #1 | osteoporosis [MeSH Terms] |
| #2 | osteopenia [MeSH Terms] |
| #3 | osteoporosis [Title/Abstract] |
| #4 | osteopenia [Title/Abstract] |
| #5 | #1 OR #2 OR #3 OR #4 |
| #6 | diabetes mellitus [MeSH Terms] |
| #7 | type 2 diabetes[MeSH Terms] |
| #8 | type 2 diabetes mellitus [MeSH Terms] |
| #9 | glycuresis [MeSH Terms] |
| #10 | diabetic [MeSH Terms] |
| #11 | diabetes [MeSH Terms] |
| #12 | diabetes mellitus [Title/Abstract] |
| #13 | type 2 diabetes [Title/Abstract] |
| #14 | type 2 diabetes mellitus [Title/Abstract] |
| #15 | glycuresis [Title/Abstract] |
| #16 | diabetic [Title/Abstract] |
| #17 | diabetes [Title/Abstract] |
| #18 | #6 OR #7 OR #8 OR #9 OR #10 OR #11 OR #12 OR #13 OR #14 OR #15 OR #16 OR #17 |
| #19 | China [MeSH Terms] |
| #20 | Chinese [MeSH Terms] |
| #21 | Mainland China [MeSH Terms] |
| #22 | China [Title/Abstract] |
| #23 | Chinese [Title/Abstract] |
| #24 | Mainland China [Title/Abstract] |
| #25 | #19 OR #20 OR #21 OR #22 OR #23 OR #24 |
| #26 | Prevalence [MeSH Terms] |
| #27 | Prevalence rate [MeSH Terms] |
| #28 | Rate [MeSH Terms] |
| #29 | Morbidity [MeSH Terms] |
| #30 | Epidemiology [MeSH Terms] |
| #31 | epidemiological investigation [MeSH Terms] |
| #32 | epidemiology investigation [MeSH Terms] |
| #33 | Epidemiology survey [MeSH Terms] |
| #34 | Epidemiological survey [MeSH Terms] |
| #35 | Survey [MeSH Terms] |
| #36 | Occurrence [MeSH Terms] |
| #37 | Epidemiological evidence [MeSH Terms] |
| #38 | Prevalence [Title/Abstract] |
| #39 | Prevalence rate [Title/Abstract] |
| #40 | Rate [Title/Abstract] |
| #41 | Morbidity [Title/Abstract] |
| #42 | Epidemiology [Title/Abstract] |
| #43 | epidemiological investigation [Title/Abstract] |
| #44 | epidemiology investigation [Title/Abstract] |
| #45 | Epidemiology survey [Title/Abstract] |
| #46 | Epidemiological survey [Title/Abstract] |
| #47 | Survey [Title/Abstract] |
| #48 | Occurrence [Title/Abstract] |
| #49 | Epidemiological evidence [Title/Abstract] |
|  | #26 OR #27 OR #28 OR #29 OR #30 OR #31 OR # OR #32 OR #33 OR #34 OR #35 OR #36 OR #37 OR #38 OR #39 OR #40 OR #41 OR #42 OR #43 OR #44 OR #45 OR #46 OR #47 OR #48 OR #49 |
| #50 | #5 OR #18 OR #25 OR #49 |

The search strategy will be modified as required for other electronic databases.
